# Yield Performance and Oil Chemotype Variation of *Coriandrum sativum* L. Genotypes under Different Sowing Dates in a Mediterranean Climate

**DOI:** 10.1021/acsomega.5c13325

**Published:** 2026-03-17

**Authors:** Dua Ahmet Ali Ali, Muzaffer Barut, Ozlem Toncer, Sengul Karaman, Leyla Sezen Tansi

**Affiliations:** † Department of Field Crops, Faculty of Agriculture, Çukurova University, Adana 01330, Turkey; ‡ Department of Field Crops, Faculty of Agriculture, 37507 Dicle University, Diyarbakir 21280, Turkey; § Department of Biology, Faculty of Science and Letter, Kahramanmaras Sutcu Imam University, Kahramanmaras 46100, Turkey

## Abstract

Coriander is a multipurpose medicinal and aromatic plant whose economic value is determined by seed yield and chemical profile of its essential and fixed oils. In the Mediterranean basin, a climate change hotspot, optimizing the cropping calendar is vital to mitigate environmental stress. This study aimed to evaluate the genotype-by-environment interaction regarding yield components and phytochemical characteristics of six coriander genotypes (Erba, Gamze, Irak, Kudret, Mardin, and Pelmus) across four sowing dates (November 01, November 15, March 01, and March 15) in the eastern Mediterranean region of Turkey. A split-plot field experiment was conducted over two consecutive growing seasons (2020–2020 and 2021–2022) in Adana. Agronomic traits were recorded, and chemical compositions of essential oils and seed oils were determined using GC-MS. The sowing date significantly influenced productivity; early autumn sowing (Nov 01) achieved the maximum seed yield (3134.63 kg/ha), while spring sowings (March) resulted in a substantial reduction of approximately 70% due to a shortened vegetative period. Genotype G1 (Erba) emerged as the most productive cultivar (2463.65 kg/ha). The essential oil content ranged from 0.52 to 1.03%, with linalool as the predominant constituent (47.44–74.96%). While early November sowings maximized the linalool content, spring sowings favored the accumulation of aliphatic aldehydes (n-decanal and 2-dodecenal). Fixed oil analysis revealed a dominance of petroselinic acid (69.93–85.82%), which exhibited a strong negative correlation with linoleic acid. PCA effectively distinguished the superior agronomic and monoterpenol-rich profiles of autumn-sown genotypes from aldehyde-rich spring sowings. To maximize both the seed yield and high-quality essential oil content in Mediterranean climates, cultivation of the “Erba” genotype during the early autumn window (early November) is highly recommended.

## Graphical abstract

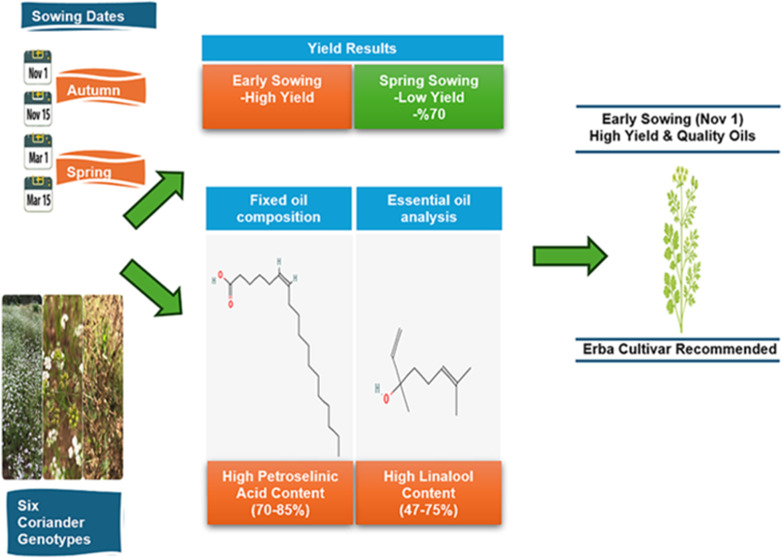

## 1 Introduction

Coriander (*Coriandrum sativum* L.), an annual herbaceous plant belonging to the Apiaceae family, is globally recognized as an important spice and medicinal crop cultivated for both its fresh aromatic foliage and dried fruits.^1–3^ In Turkey, the species *C. sativum* and *Coriandrum tordylium* grow naturally. *C. sativum* is found at elevations of 320–1300 m in northwestern, southern, and eastern Anatolia, while *C. tordylium* grows at around 900 m in Central Anatolia, Lebanon, and the Syrian Desert.^4^ Although coriander production in Turkey fluctuates, according to the 2024 data, Burdur is the most predominant production center. In that year, 43 tons were produced on 115.2 ha of land, accounting for 63% of Turkey’s coriander total cultivation area and 43% of its production.^5^ Beyond its regional importance, the crop’s industrial versatility is dictated by its diverse biochemical profile, rendering it a crucial raw material in the food, cosmetic, and pharmaceutical industries.^6–8^

The economic value of the crop is largely derived from its essential oil, in which linalool is the predominant monoterpene, and its fatty oil, characterized by a high concentration of petroselinic acid utilized in various industrial applications.^9–11^ While the biosynthesis of these secondary metabolites and the accumulation of biomass are highly sensitive to variations in environmental conditions and agronomic management,^12–14^ there is a lack of integrated knowledge on how extreme thermal shifts specifically alter the partition between these two oil types. Abiotic factors, particularly temperature, humidity, and nutrient availability, play a critical role in defining the crop’s physiological performance and final yield.^15–17^ Consequently, identifying optimal agronomic practices is essential for exploiting the full genetic potential of the crop to achieve maximum seed and oil yields.^18,19^ However, traditional practices are increasingly challenged by rapid climatic shifts.

Among agronomic factors, the determination of an appropriate sowing date is paramount as it dictates the specific climatic regime the plant experiences throughout its phenological development.^20–22^ The sowing date significantly influences the duration of the vegetative phase and the transition to the reproductive stage.^23^ The literature indicates that delaying the sowing date often exposes the crop to higher temperatures during critical growth stages, shortens the vegetative period, and forces plants to mature earlier.^24–27^ Conversely, optimizing the sowing window ensures that flowering and grain filling coincide with favorable temperature and photoperiodic conditions, thereby maximizing the seed yield and quality.^28–30^

However, the response to these environmental constraints is not uniform across the species; rather, it is governed by a significant genotype-by-environment interaction.^31–33^ Different genotypes exhibit varying degrees of phenotypic plasticity and stability regarding yield components and essential oil profiles when subjected to diverse climatic conditions.^34–36^ For instance, certain cultivars may demonstrate superior resilience to heat stress or photoperiodic changes, maintaining higher essential oil yields even under suboptimal sowing conditions.^37–39^ Therefore, selecting suitable genotypes that are specifically adapted to local climatic zones is a prerequisite for maximizing the productivity.

Although coriander originates from the Mediterranean region, this area is currently identified as a hotspot for climate change, facing increasing temperatures and irregular precipitation patterns that pose significant challenges to agricultural sustainability warming at a rate 20% faster than the global average.^40,41^ These unpredictable weather patterns and increasing temperatures necessitate a reevaluation of traditional cropping calendars to mitigate yield losses and maintain biochemical quality.^7,42^ The success of the crop under such shifting conditions is governed by a significant Genotype-by-Environment (G × E) interaction, which refers to the nonuniform response of different genotypes to varying climatic regimes, such as temperature fluctuations and photoperiodic changes, dictating the phenotypic plasticity and chemical stability of the plant. Despite extensive research on yield parameters, the specific mechanism by which G × E interactions modulate the chemical transition from monoterpenes to aliphatic aldehydes remains largely unexplored. This study addresses this critical gap by providing a comprehensive analysis of the metabolic shift between monoterpenes and aliphatic aldehydes under varying environmental stress. The primary novelty of this research lies in moving beyond simple yield observations to establish a dual-purpose production model. By characterizing the divergent metabolic pathways of both essential and fixed oils triggered by heat stress in the Eastern Mediterranean context, this work offers a new biochemical framework for maintaining the oil quality under the pressures of climate change.

## 2 Materials and Methods

### 2.1 Plant Material and Field Experiment

Field trials were conducted during the 2020–2021 and 2021–2022 growing seasons at the experimental station of Çukurova University in Adana, Turkey (37°00′55.30″N, 35°21′26.30″E). The study area is characterized by a Mediterranean climate (Figure 1). Monthly averages of daily evapotranspiration (*ET*_*o*_), relative humidity, wind speed, and soil temperature during the 2020–2021 and 2021–2022 growing seasons compared to the long-term (1995–2022) climatic averages at the experimental site (Table S1). The soil, classified as clay-loam with low organic matter (1.10%), was prepared by deep plowing (30 × 40 cm), followed by cultivation. The experiment was arranged in a split-plot design with three replications. Four sowing dates (November 01, November 15, March 01, and March 15) were assigned to the main plots, while six coriander genotypes comprising four registered cultivars (Erba, Gamze, Kudret, and Pelmus) and two local landraces (Irak and Mardin populations) were used and allocated to the subplots. Seeds were sourced from Dicle University (Prof. Ozlem Toncer) and a local producer in Duhok. Each subplot (3.6 m^2^) consisted of four rows, with seeds sown at a depth of 1–2 cm. Fertilization included 25 kg/ha of N and P_2_O_5_ via diammonium phosphate (DAP); no potassium was applied due to high indigenous soil levels. Weeds were managed manually without the use of pesticides. At maturity—signified by brown pods and black seeds in early June—ten plants were randomly selected from the middle rows of each plot and pooled to provide a representative composite sample for analysis. Uniform irrigation was maintained across all treatments to ensure nonlimiting moisture conditions. The timing was determined using a manual soil moisture probe method, where soil core samples were taken regularly from 0 to 30 cm depth. Irrigation was applied when the soil reached a state of perceptible dryness, ensuring that the moisture remained near field capacity. The growing degree days (GDD) (Table S2) were calculated based on the daily temperature data following the method suggested by Iwata (1984)^43^

$$\mathrm{GDD}=\Sigma \left[\frac{T_{\max}+T_{\min}}{2}-T_{b}\right]$$

where *T*_max_ and *T*_min_ are the daily maximum and minimum temperatures (°C), respectively. The base temperature (*T*_b_) was taken as 4.8 °C, as established for coriander by Hernandez-Devi et al. (2002)^44^

**Figure 1:**
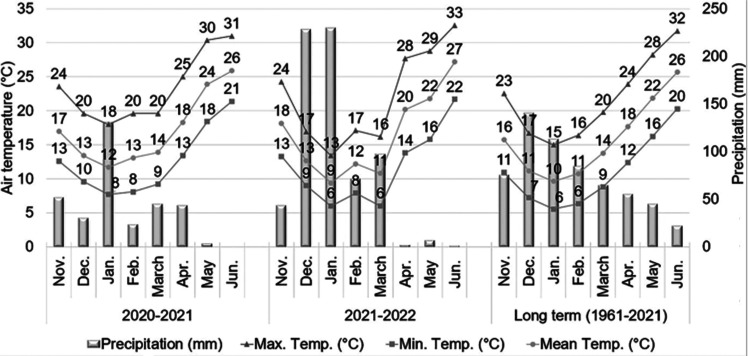
Comparative meteorological data for the study duration and historical period (1961–2021). All climatic records were provided by the Adana Meteorological Directorate.

The heat use efficiency (HUE) for seed yield was determined according to the procedure described by Pal and Murty (2010)^45^

$$\mathrm{HUE}=\frac{s\mathrm{eed}\;\mathrm{yield}\;\left(\mathrm{kg}/\mathrm{ha}\right)}{\sum \mathrm{HU}}$$

where ∑HU is the cumulative heat unit (°C day). The resulting HUE values are expressed in kg seed/ha/deg. day. These meteorological parameters were recorded at the weather station of the Adana Meteorological Directorate, consistent with the methodology described by Moniruzzaman et al. (2015).^46^

### 2.2 Essential Oil Extraction

Essential oils were extracted from 30 g of ground coriander seeds via hydrodistillation using a Clevenger apparatus^47^ for 3 h with 300 mL of distilled water. Following extraction, the essential oil content was measured and expressed as a weight-to-weight percentage (w/w) based on the seed dry matter. All extractions and subsequent GC-MS analyses were performed on three independent biological replicates to ensure statistical validity.

### 2.3 Seed Oil Extraction

Coriander seed oil was obtained through an ultrasound-assisted extraction (UAE). A 5 g sample of pulverized seeds was processed with 117 mL of *n*-hexane at 55 °C for 45 min. Following extraction, the solvent was removed via rotary evaporation at 70 °C. The resulting oil mass was determined gravimetrically at the Department of Field Crops, Faculty of Agriculture, Çukurova University.^48^

### 2.4 Gas Chromatography/Mass Spectrometry (GC/MS) Analysis

The chemical profiles of the essential oil (EO) and fatty acids were characterized at the Department of Biology, Kahramanmaraş Sütçü İmam University, following the methodology of Toncer et al. (2022).^49^ For EO analysis, 10 μL of sample was diluted in 250 μL of dichloromethane and a 1 μL aliquot was injected into an Agilent 6890 II GC coupled with an Agilent 5975C mass spectrometer. Chromatographic separation was performed on an HP-88 capillary column (100 × 250 × 0.20 μm) using helium as the carrier gas (1.0 mL/min). The oven temperature program began at 70 °C (1 min), increased to 220 °C at 10 °C/min (10 min hold), and finally reached 230 °C at 10 °C/min (10 min hold). The injector temperature was maintained at 250 °C with a split ratio of 20:1. Mass spectra were acquired in electron impact (EI) mode at 70 eV (*m*/*z* = 35–400) and identified via comparison with Flavor2, HPCH1607, and Wiley7Nist05 databases. For seed oil analysis, fatty acids were first transmethylated using 0.5 mL of 2 N KOH–CH_3_OH. The resulting methyl esters were analyzed using the same GC-MS system with a modified oven program: 170 °C (1 min), rising to 220 °C at 10 °C/min, and finishing at 230 °C (15 min hold).

### 2.5 Statistical Analysis

Agronomic data were subjected to analysis of variance (ANOVA) based on a split-plot experimental design, where sowing dates and genotypes were assigned as the main plot and subplot factors, respectively. These analyses, along with a correlation-based principal component analysis (PCA), were conducted by using JMP software (version 14.0, SAS Institute Inc.). Mean separation was performed using Tukey’s Honestly Significant Difference (HSD) test at a significance level of *P* ≤ 0.05. Furthermore, Pearson correlations was visualized in Python using Seaborn/Matplotlib and R software using Metan, while Flourish Studio was employed for heat map visualization.

## 3 Results and Discussion

### 3.1 Agronomic Traits

Analysis of variance (ANOVA) revealed significant differences among years, sowing dates, and different coriander genotypes for the majority of the evaluated agronomic traits (Table 1). As shown in Figure 1, the 2021–2022 growing season received more consistent precipitation during the critical early vegetative and preflowering stages in January and February compared to 2020–2021. This enhanced moisture availability, combined with the absence of extreme temperature dips during flowering, likely facilitated the increase in plant height and the significantly higher reproductive output observed in the second year. The correlation analysis demonstrates that Heat Use Efficiency (HUE) and Growing Degree Days (GDD) are the primary climatic drivers of productivity, showing strong positive correlations with seed yield (Figure S1). Furthermore, while precipitation significantly contributes to both yield and vegetative growth, humidity exhibits nonsignificant relationships across all measured parameters, indicating it was not a limiting factor for development in this study.

**Table 1:** Analysis of Variance (ANOVA) and Mean Separation of Yield and Yield-Related Components in Coriander as Influenced by the Year, Sowing Date, Genotype, and Their Interactions^a^

factors	plant height (cm)	number of branches (per plant)	number of umbels (per plant)	number of umbellets (per plant)	number of seeds (per plant)	seed weight (g/plant)	thousand seed weight (g)	seed yield (kg/ha)
years (y)								
2021	68.00b	5.50	36.12b	156.56b	400.08b	2.43b	6.61a	971.59b
2022	75.98a	5.69	93.99a	494.09a	1174.72a	8.04a	6.08b	3214.16a
sowing dates (s)								
01 Nov	98.04a	5.80a	92.87a	445.37a	1120.63a	7.84a	6.43a	3134.63a
15 Nov	92.95b	5.73a,b	88.13a	443.04a	1138.84a	7.55a	6.47a	3018.90a
01 March	49.87c	5.34b	42.95b	224.12b	522.50b	3.33b	6.57a	1332.13b
15 March	47.11c	5.50a,b	36.27b	188.77b	367.63c	2.21c	5.91b	885.85c
genotypes (g)								
G1	81.03a	6.16a	83.57a	420.16a	1019.80a	6.16a	6.21d	2463.65a
G2	65.60b	5.56b	67.99a,b	333.72b	760.21b,c	5.04a,b	6.84b,c	2017.08a,b
G3	57.02c	4.89c	43.18c	205.68c	514.91d	4.80b	8.37a	1919.59b
G4	80.13a	5.77a,b	67.26b	339.35a,b	894.60a,b	5.44a,b	3.39e	2177.95a,b
G5	70.41b	5.68a,b	62.22b	316.73b	673.16c,d	4.79b	6.95b	1917.00b
G6	77.75a	5.50b	66.12b	336.31b	861.72a,b	5.15a,b	6.33c,d	2061.98a,b
**mean**	**71.99**	**5.59**	**65.05**	**325.32**	**787.40**	**5.23**	**6.35**	**2092.88**
Tukey_Y_ (% 5)	2.10**	ns	6.41**	32.35**	68.86**	0.52**	0.24**	208.70**
Tukey_S_ (% 5)	3.92**	0.42*	11.96**	60.33**	128.44**	0.97**	0.45**	389.30**
Tukey_G_ (% 5)	5.34**	0.57**	16.29**	82.22**	175.03**	1.33*	0.61**	530.50*
Tukey_S × G_ (% 5)	13.76**	1.47**	ns	ns	ns	ns	1.57**	ns

a RT: Retention time, G1: Erba, G2: Gamze, G3: Irak, G4: Kudret, G5: Mardin, G6: Pelmus, ns: not significant, *P < 0.05, **P < 0.01, *Levels not connected by the same letter are significantly (*P* < 0.05) different according to the Tukey’s test. Values for sowing dates and genotypes represent the mean of the 2020–2021 and 2021–2022 seasons.

Plant height was significantly influenced by all of the main factors, including the year, sowing date, and genotype. Plants grown in the 2021–2022 season were significantly taller (75.98 cm) than those in 2020–2021 (68.00 cm), a 12% increase likely due to more favorable climatic conditions. Sowing date exerted a profound effect; early autumn sowing (01 Nov) resulted in a maximum plant height (98.04 cm), while late spring sowings (March) caused a reduction of approximately 50%. This decline is attributed to the shortened vegetative period in spring sowing, which accelerates the transition to the reproductive stage. Among the genotypes, G1 (81.03 cm) and G4 (80.13 cm) were the tallest. Our findings align with the variation reported by Kaium (2013)^50^ and Katar et al. (2016),^33^ although they remain lower than the maximum heights reported under different ecological conditions by Rashed and Darwesh (2015)^7^ and Uikey (2022).^51^ These variations confirm that earlier establishment allows for superior vegetative development, consistent with the conclusions of Kassu et al. (2018).^53^

While the effect of the year on the number of branches was statistically nonsignificant, both sowing dates and genotypes showed significant differences. The highest branching capacity was recorded in the 01 Nov sowing (5.80 branches/plant), with a significantly decreasing trend as sowing was delayed. Genotypically, G1 exhibited the highest capacity (6.16 branches/plant). Notably, a significant Sowing × Genotype interaction indicates that the lateral branching response is dependent on the sowing window. These results corroborate the findings of Katar et al. (2016),^33^ Rai et al. (2020),^39^ and Sharangi and Roychowdhury (2014).^22^ The lower values compared to Kamal et al. (2023)^42^ (up to 17.33 branches) are likely attributable to differences in environmental conditions or the specific genetic material used.

Year, sowing date, and genotype had a highly significant impact on the number of umbels per plant. The 2021–2022 season was far superior, producing nearly 2.5 times more umbels (93.99) than 2020–2021. Similarly, November sowings optimized reproductive output, while March sowings resulted in a substantial reduction. Genotype G1 demonstrated superior performance (83.57 umbels/plant), exceeding the ranges reported by Uikey (2022),^51^ Kamal et al. (2023),^42^ and Katar et al. (2016).^33^ Regarding umbellets per plant, values in 2021–2022 reached 494.09, tripling the previous year’s average. The total reproductive capacity observed in G1 (420.16 umbellets/plant) is considerably higher than that reported by Diwan et al. (2018).^54^ While most literature focuses on umbellets per umbel, these results highlight the substantial total reproductive potential of early sown genotypes under favorable Mediterranean conditions.

The number of seeds per plant followed a trend similar to other yield components, where early sowings (01 and 15 Nov) established clear superiority. Genotype G1 produced the highest number of seeds (1019.80), which is highly consistent with the upper limits reported by Kaium (2013)^50^ and exceeds the values found by Rai et al. (2020)^39^ and Delibaltova et al. (2012).^55^

Seed weight per plant was significantly higher in 2021–2022 (8.04 g/plant) compared to 2020–2021. The 01 Nov and 15 Nov dates were the most productive, whereas yield dropped to 2.21 g/plant in mid-March. Genotype G1 (6.16 g/plant) was the most efficient in terms of mass accumulation. Our results are comparable to Sharangi and Roychowdhury (2014)^22^ but remain lower than the exceptionally high yields reported by Rashed and Darwesh (2015).^7^

Interestingly, Thousand Seed Weight (TSW) exhibited a compensatory mechanism; TSW was significantly higher in 2020–2021 (6.61 g) than in 2021–2022 (6.08 g). The lower seed number in the first year likely allowed for greater photosynthetic allocation to individual grains. G3 achieved the highest TSW (8.37 g), confirming that this trait is highly genotype-dependent and often inversely correlated with total seed number, as supported by Katar et al. (2016)^33^ and Diwan et al. (2018).^54^

Total seed yield per hectare revealed the most striking differences (Figure 2). Yield was higher in 2021–2022 (3214.16 kg/ha) than in 2020–2021 (971.59 kg/ha), emphasizing the critical role of annual climatic variations. The 01 Nov sowing provided the maximum yield (3134.63 kg/ha), while delaying to 15 March resulted in a minimum yield (885.85 kg/ha). G1 emerged as the most promising cultivar (2463.65 kg/ha). The peak yield of G1 aligns with the upper results of Delibaltova (2020)^56^ and Kassu et al. (2018).^53^ Favorable environmental conditions, combined with early sowing and superior genotype selection, significantly enhanced performance, whereas late sowing resulted in drastic reductions regardless of the genotype.

**Figure 2:**
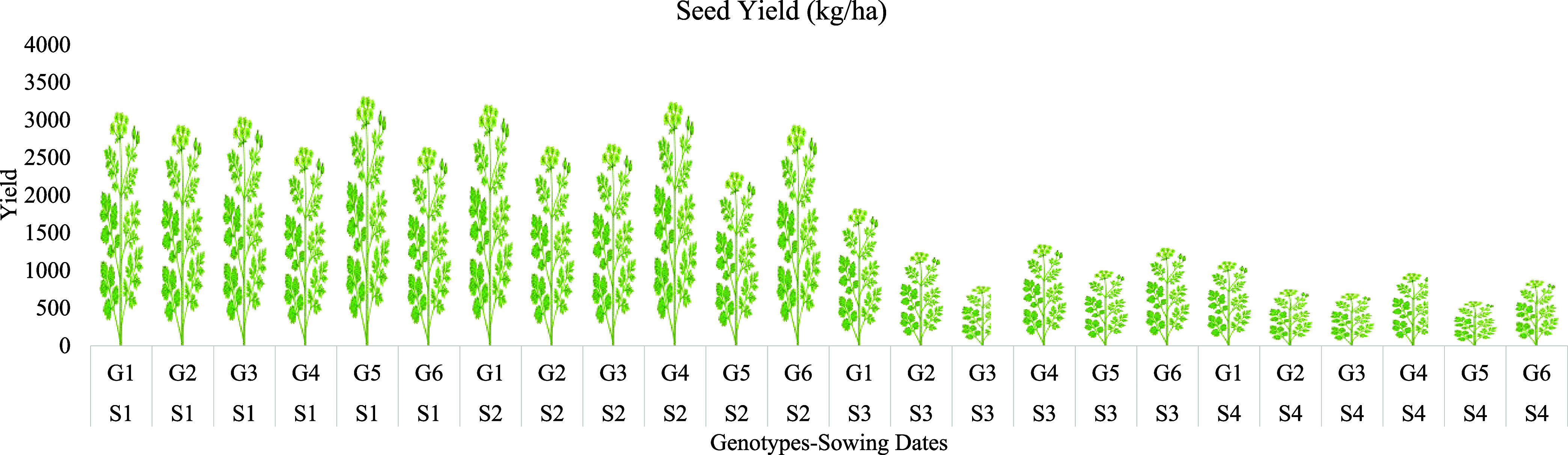
Seed yield performance of different genotypes (kg/ha) (average of two growing seasons) (S1: 01 Nov, S2: 15 Nov, S3: 01 March, S4: 15 March, G1: Erba, G2: Gamze, G3: Irak, G4: Kudret, G5: Mardin, G6: Pelmus).

### 3.2 Essential Oil Content and Composition

The essential oil (EO) content and the relative proportions of its chemical constituents showed variations influenced by both the genotype and sowing date (Table 2). The EO content of the coriander genotypes across different sowing dates exhibited a range between 0.52% (G2, 15 Nov) and 1.03% (G4, 01 Nov). Our findings align well with the higher ranges documented by Zheljazkov et al. (2008)^21^ (0.80–2.20%) and Delibaltova (2020)^56^ (0.61–1.17%) while proving notably higher than the relatively low yields observed by Rashed and Darwesh (2015)^7^ (0.21–0.38%), Kamal et al. (2023)^42^ (0.14–0.35%), and Izgi (2020)^17^ (0.16–0.21%). In the autumn sowings, the oil content for the 01 Nov date was generally higher, ranging from 0.61 to 1.03%, while it varied between 0.52 and 0.82% for the 15 Nov date. During the spring sowing periods, the values were observed to be between 0.68 and 0.98% for 01 March, and between 0.58 and 0.81% for 15 March. These results, supported by the 0.329–1.3% range reported by Uikey et al. (2022; 2023)^51,52^ and Rai et al. (2020)^39^ indicate that total oil accumulation is highly sensitive to both the genetic background and timing of the sowing.

**Table 2:** Essential Oil Composition of Coriander Based on the Different Sowing Dates and Genotypes^a^

sowing dates		01 Nov						15 Nov					
genotypes		G1	G2	G3	G4	G5	G6	G1	G2	G3	G4	G5	G6
essential oil content (%)		0.91	0.76	0.61	1.03	0.76	0.84	0.80	0.52	0.67	0.78	0.62	0.82
compounds	RT (min)	relative peak area (%)											
β-pinene	11.465	0.31	0.33	0.21	0.33	0.24	0.42	0.45	0.37	0.34	0.37	0.32	0.40
myrcene	11.649	0.58	0.62	0.41	0.44	0.28	0.70	0.78	0.69	0.66	0.61	0.57	0.50
limonene	12.142	1.07	1.08	0.97	0.64	0.49	0.77	1.41	1.40	1.25	1.17	1.21	1.03
γ-terpinene	12.676	1.65	3.81	2.67	1.30	1.94	5.06	4.45	3.50	2.05	3.04	3.10	2.06
α-terpinene	13.052	0.08	0.27	0.12	0.06	0.07	0.33	0.38	0.18	0.07	0.27	0.23	0.20
heptanal	13.443	nd	0.09	nd	nd	0.10	nd	0.10	nd	nd	nd	0.07	0.09
p-cymene	13.620	4.80	5.04	3.04	2.86	2.31	3.51	3.63	3.67	3.93	3.96	4.37	2.39
octanal	14.749	nd	0.12	0.10	0.09	0.27	0.06	0.13	nd	nd	0.18	0.21	0.23
nonanal	16.102	nd	0.12	0.20	0.14	0.16	0.10	0.14	nd	0.17	0.08	0.17	0.03
2-octylfuran	16.215	nd	0.10	0.14	nd	0.13	nd	0.11	nd	nd	nd	0.09	nd
linalool oxide B	16.310	0.22	0.20	0.29	0.41	0.15	0.22	0.19	nd	0.34	0.20	0.25	nd
linalool	16.938	74.96	59.11	60.79	67.33	54.97	68.06	59.57	66.62	59.49	63.21	50.64	52.81
n-decanal	17.455	2.39	4.55	5.89	4.82	7.31	3.89	4.76	3.09	4.60	3.90	6.67	5.35
2-decylfuran	18.589	nd	0.23	0.26	0.26	0.12	0.14	0.21	nd	nd	0.07	0.28	nd
undecanal	18.714	nd	0.39	0.43	0.43	0.30	0.25	0.28	nd	nd	nd	0.36	0.10
isoborneol	19.509	nd	2.97	2.92	nd	nd	nd	2.60	nd	nd	nd	nd	nd
camphor	19.525	2.20	nd	nd	1.44	2.06	1.49	nd	2.32	2.33	1.99	2.91	2.41
cis-geraniol	19.711	6.61	5.22	5.00	5.58	3.08	5.45	6.20	4.77	4.84	7.25	4.66	4.81
trans-2-octenal	20.097	2.11	8.20	8.51	5.48	14.60	3.16	8.56	5.70	8.85	6.11	10.96	15.17
trans-2-undecen-1-ol	20.856	1.59	1.29	1.08	1.00	0.68	0.87	1.10	1.26	0.98	0.81	1.48	0.78
trans-2-tridecen-1-ol	21.248	nd	0.49	0.49	0.50	0.68	0.26	0.35	0.45	0.55	0.37	0.72	0.78
2-dodecenal	22.334	0.92	4.04	4.49	3.98	6.38	2.70	3.48	4.20	6.01	4.00	6.12	7.45
tridecen-1-al	24.388	0.45	1.62	1.97	2.40	3.42	2.09	1.13	1.77	3.54	1.87	3.36	3.41

sowing dates		01 March						15 March					
genotypes		G1	G2	G3	G4	G5	G6	G1	G2	G3	G4	G5	G6
essential oil content (%)		0.80	0.75	0.68	0.92	0.90	0.98	0.80	0.81	0.58	0.67	0.68	0.75
compounds	RT (min)	relative peak area (%)											
β-pinene	11.465	0.23	0.38	0.27	0.24	nd	0.21	0.53	0.41	0.33	0.39	0.35	0.43
myrcene	11.649	0.29	0.46	0.52	0.36	nd	0.29	0.57	0.62	0.52	0.59	0.68	0.72
limonene	12.142	0.59	0.88	0.84	0.87	nd	0.63	1.09	1.04	0.83	1.03	1.14	1.35
γ-terpinene	12.676	1.29	1.27	2.84	0.97	3.14	0.75	2.78	4.41	3.53	2.65	3.53	2.17
α-terpinene	13.052	nd	nd	0.08	nd	nd	nd	0.24	0.34	0.23	0.24	0.27	0.26
heptanal	13.443	nd	nd	0.06	nd	nd	nd	nd	0.20	0.14	nd	0.10	0.06
p-cymene	13.620	1.85	3.33	6.46	2.78	3.61	2.59	2.52	2.89	3.87	2.67	4.50	2.49
octanal	14.749	0.30	0.25	0.26	0.32	nd	0.39	0.11	0.20	0.19	0.10	0.27	0.17
nonanal	16.102	0.08	0.12	0.16	0.09	nd	0.24	0.10	0.05	0.26	0.14	0.20	0.14
2-octylfuran	16.215	nd	nd	0.12	nd	nd	0.04	0.12	0.04	0.25	0.27	0.17	0.09
linalool oxide B	16.310	nd	nd	0.21	nd	nd	0.44	0.13	nd	0.28	nd	0.28	0.26
linalool	16.938	67.80	56.09	54.65	69.06	53.94	55.74	53.70	65.46	47.44	57.85	50.52	56.19
n-decanal	17.455	4.55	6.81	6.11	3.87	9.56	5.72	5.82	4.83	6.85	5.74	6.68	5.73
2-decylfuran	18.589	nd	nd	0.28	nd	nd	nd	nd	0.26	0.27	0.18	0.24	0.18
undecanal	18.714	nd	nd	0.43	nd	nd	nd	0.23	0.47	0.73	nd	nd	0.31
isoborneol	19.509	2.26	4.11	nd	2.81	2.39	nd	2.20	2.17	nd	2.51	3.78	2.90
camphor	19.525	nd	nd	1.70	nd	nd	2.43	nd	nd	2.74	nd	nd	nd
cis-geraniol	19.711	4.43	5.62	4.53	5.53	2.82	4.53	5.55	5.51	4.58	5.75	4.84	5.83
trans-2-octenal	20.097	7.14	9.98	7.28	6.23	16.64	13.83	9.31	6.72	12.60	7.09	10.45	7.94
trans-2-undecen-1-ol	20.856	1.22	0.66	0.86	1.32	nd	1.02	1.17	1.01	0.90	1.16	1.47	1.21
trans-2-tridecen-1-ol	21.248	0.27	0.46	0.64	0.36	nd	0.49	0.56	0.24	0.75	0.53	0.55	0.55
2-dodecenal	22.334	3.92	5.23	5.69	3.12	5.60	6.06	8.55	1.97	6.79	6.85	4.79	6.34
tridecen-1-al	24.388	3.11	3.73	3.59	2.05	2.31	4.34	3.89	1.17	3.86	3.25	2.69	3.80

a RT: Retention time, G1: Erba, G2: Gamze, G3: Irak, G4: Kudret, G5: Mardin, G6: Pelmus. Means are average of two growing seasons.

Regarding the chemical composition of the seed oil, linalool remained the predominant constituent across all samples, consistent with reviewed literature where it typically accounts for more than half of the total oil volume. In this study, the relative area of linalool ranged from 47.44% (G3, 15 March) to 74.96% (G1, 01 Nov). These findings agree with the high linalool concentrations reported by Rashed and Darwesh (2015)^7^ (65.1–89.41%) and Msaada et al. (2009)^9^ (80–87% in mature seed), as well as the broader ranges documented by Laribi et al. (2015)^3^ (40–87.5%) and Kamal et al. (2023)^42^ (51.4–79.8%). The highest linalool levels in our study were consistently observed on the Nov 01, 2015 sowing date, where five out of six genotypes exceeded 59%. In contrast, the March 15 sowing date resulted in lower linalool proportions, varying between 47.44 and 65.46%. Other characteristic secondary metabolites identified included γ-terpinene and p-cymene, which also showed variation; γ-terpinene ranged from 0.75% (G6, 01 March) to 5.06% (G6, 01 Nov), while p-cymene levels fluctuated between 1.85% (G1, 01 March) and 6.46% (G3, 01 March). These variations are comparable to the significant minor components identified by Delibaltova (2020),^56^ such as camphor (4.0–8.4%) and γ-terpinene (5.0–7.5%), and the geranyl acetate levels reported by Izgi (2020)^17^ and Kamal et al. (2023).^42^

A distinct differentiation in the volatile profile was observed in the aliphatic aldehyde fraction, which is critical for the aroma profile of the coriander. In our study, the relative area of n-decanal ranged from 2.39% (G1, 01 Nov) to 9.56% (G5, 01 March), while trans-2-octenal exhibited a broad range from 2.11% (G1, 01 Nov) to a maximum of 16.64% (G5, 01 March). Furthermore, 2-dodecenal values were found to be between 0.92% (G1, 01 Nov) and 8.55% (G1, 15 March). It was observed that genotypes such as G5 and G6 tended to accumulate higher proportions of these aldehydes during the spring sowing dates compared to those during the autumn periods. In the literature, El-Zaeddi et al. (2020)^57^ demonstrated that coriander leaf oil is particularly rich in aliphatic aldehydes, such as decanal, E-2-dodecenal, 1-decanol, and dodecanal, whereas seed oils are typically characterized by monoterpenols. This increase in aldehydes suggests a stress-induced metabolic reprogramming where heat stress during the shortened phenological cycle likely leads to the premature termination of the linalool biosynthesis pathway.^57^ In other words, as temperatures rise in the late spring months of the Mediterranean basin, the shortened vegetative period likely limits the full expression of the linalool biosynthesis pathway. This thermal stress may trigger the oxidative degradation of membrane lipids, leading to a higher concentration of aldehydes as a secondary defense or breakdown mechanism. This shift confirms that while autumn sowings maximize monoterpene quality, spring conditions alter the enzymatic environment, favoring fatty acid oxidation products. Overall, the chemical profile was characterized by a dominance of linalool, supplemented by variations in the aldehyde and monoterpene fractions. While the 01 Nov sowing date favored the highest linalool and EO content, the 01 March sowing date led to an increase in aldehydes, particularly in specific genotypes like G5. From a practical standpoint, the high stability of genotype G1 (Erba) across the November sowing dates suggests that it is a robust candidate for large-scale cultivation. Its ability to maintain superior seed yield (2463.65 kg/ha) and peak linalool levels under varying early season conditions provides a reliable safety margin for producers. These findings demonstrate that the chemical diversity of coriander essential oil is strongly influenced by the interaction between the sowing date and genotype, providing a wide range of chemical profiles for different industrial and pharmacological purposes.

### 3.3 Fixed Oil Content and Fatty Acid Composition

The results of this study regarding the fixed oil content and fatty acid composition of coriander genotypes demonstrate variability influenced by sowing dates and environmental conditions (Table 3). The oil content of the seeds was observed in a range between 10.4 and 17.87% for the 01 Nov sowing, while these values shifted to a range of 7.66–17.5% in the 15 Nov sowing. As the sowing dates progressed into the spring, the oil content values were recorded between 9.16 and 16.12% for 01 March, eventually settling within a narrower range of 8.42–10.2% for the 15 March sowing. In comparison, a review of the literature reveals significant variations in fixed oil ratios depending on the researchers and study conditions; for instance, Izgi^17^ reported higher values ranging between 22.74 and 23.94%, while Laribi et al.^3^ observed a broader range of 19.24–28.4%. These differences highlight the substantial influence of genetic factors and environmental conditions on lipid accumulation.

**Table 3:** Fatty Acid Composition of Coriander Based on Different Sowing Dates and Genotypes^a^

sowing dates		01 Nov						15 Nov					
genotypes		G1	G2	G3	G4	G5	G6	G1	G2	G3	G4	G5	G6
oil content (%)		17.87	13.78	10.40	15.78	11.00	16.95	17.50	12.75	7.66	14.50	9.19	15.75
compounds	RT (min)	relative peak area (%)											
C12:0 lauric acid	10.688	nd	nd	nd	0.13	nd	0.04	nd	nd	0.22	0.05	0.28	0.03
C14:0 myristic acid	11.888	0.08	0.13	0.15	0.16	nd	0.12	nd	nd	0.11	0.10	nd	0.15
C16:0 palmitic acid	13.488	6.91	6.54	7.47	8.62	4.78	5.32	9.83	7.11	6.7	6.63	9.48	7.51
C16:1 palmitoleic acid	14.288	0.53	0.40	0.55	0.58	nd	0.47	nd	nd	1.05	0.86	nd	0.6
C18:0 stearic acid	15.688	1.67	1.01	1.27	2.2	1.08	0.81	nd	nd	0.74	2.80	2.09	1.53
C18:1 petroselinic acid	16.488	84.63	84.52	83.57	83.69	78.99	78.58	83.79	85.82	81.38	82.05	83.65	80.59
C18:2 linoleic acid	17.688	5.5	6.12	6.11	2.77	13.32	11.58	3.56	6.07	8.52	6.76	2.8	8.52
C18:3 linolenic acid	19.088	nd	0.28	nd	0.34	nd	0.44	nd	nd	nd	nd	nd	0.34
C20:1 gondoic acid	19.288	0.11	nd	0.13	nd	nd	nd	nd	nd	0.38	nd	nd	0.16

sowing dates		01 March						15 March					
genotypes		G1	G2	G3	G4	G5	G6	G1	G2	G3	G4	G5	G6
oil content (%)		16.12	13.00	9.16	14.31	10.05	12.52	9.17	9.98	10.20	8.42	8.87	9.41
compounds	RT (min)	relative peak area (%)											
C12:0 lauric acid	10.688	nd	0.11	0.22	nd	nd	nd	nd	0.11	nd	nd	nd	0.02
C14:0 myristic acid	11.888	0.08	0.17	0.13	nd	0.16	nd	nd	0.15	nd	nd	1.32	0.17
C16:0 palmitic acid	13.488	9.26	8.2	6.53	10.06	5.75	10.83	11.03	7.18	5.42	12.08	5.15	6.37
C16:1 palmitoleic acid	14.288	0.53	0.64	0.8	nd	0.94	nd	nd	0.34	nd	nd	nd	0.73
C18:0 stearic acid	15.688	1.67	1.46	0.91	nd	1.31	nd	nd	0.81	0.72	nd	0.85	2.24
C18:1 petroselinic acid	16.488	83.8	83.1	79.85	82.04	69.93	83.79	84.01	83.78	75.81	81.2	76.46	78.83
C18:2 linoleic acid	17.688	4.53	5.98	10.47	5.89	18.13	5.38	4.58	5.96	16.79	5.82	12.81	8.6
C18:3 linolenic acid	19.088	nd	nd	0.54	nd	0.89	nd	nd	0.27	0.54	nd	nd	0.23
C20:1 gondoic acid	19.288	0.11	nd	0.26	nd	nd	nd	nd	nd	nd	nd	nd	0.16

a RT: Retention time, G1: Erba, G2: Gamze, G3: Irak, G4: Kudret, G5: Mardin, G6: Pelmus. Means are average of two growing seasons.

Regarding the chemical profile, the fatty acid composition was predominantly characterized by Monounsaturated Fatty Acids (MUFA), with petroselinic acid (C18:1) serving as the primary component across all samples. Its relative peak area percentage fluctuated between 69.93 and 85.82%, reinforcing the status of coriander oil as a highly monounsaturated lipid source. Other MUFA constituents, such as palmitoleic acid (C16:1) and gondoic acid (C20:1), were identified in minor or trace concentrations, ranging from 0 to 1.05% and from 0 to 0.38%, respectively. This dominance of petroselinic acid as the primary MUFA is corroborated by all studies that provided chemical data, although the specific ranges vary. Msaada et al.^9^ reported a wide variation in petroselinic acid levels (40.2–81.4%), whereas Laribi et al.^3^ and Izgi^17^ found ranges of 65.7–80.9 and 57.79–62.96%, respectively.

The Polyunsaturated Fatty Acid (PUFA) fraction in our study was mainly represented by linoleic acid (C18:2), which showed a broad distribution range from 2.77 to 18.13% across different genotypes and environmental conditions. Linolenic acid (C18:3), another PUFA member, remained at trace levels with values ranging between nd and 0.89%. Notably, the findings of Izgi^17^ differ from other researchers and the results of this study by identifying butyric acid and linolenic acid (9.51–10.53%) as major constituents. In terms of the Saturated Fatty Acid (SFA) profile, palmitic acid (C16:0) was the major contributor with concentrations between 4.78 and 12.08%, while stearic acid (C18:0) levels remained between nd and 2.8%. Other saturated components, specifically lauric acid (C12:0) and myristic acid (C14:0), were observed in relatively small amounts, with ranges of 0–0.28% and 0–1.32%, respectively.

Overall, the core fatty acid profile is characterized by a high degree of monounsaturation (high MUFA/PUFA ratio) dominated by petroselinic acid regardless of the sowing time. This suggests that while environmental factors significantly impact the total oil yield, the underlying MUFA-rich chemical skeleton remains relatively stable. Furthermore, it is observed that many researchers, including Rashed and Darwesh,^7^ Zheljazkov et al.,^21^ and Kamal et al.,^42^ did not specify fixed oil parameters in their results, suggesting that their studies may have focused primarily on essential oils or other agronomic characteristics, which further highlights the importance of the lipid profiles established in this research.

The chemical characterization and the influence of agronomic variables on the plant’s profile are clearly illustrated in Figures 3 and 4. According to the representative GC/MS chromatograms presented in Figure 3, linalool was identified as the predominant essential oil component (a), while C18:1 petroselinic acid emerged as the major fatty acid (b) within the fixed oil profile. The quantitative distribution and variation of these primary markers across different genotypes and sowing dates are further elucidated in the heat map analysis in Figure 4. The data indicate a significant interaction between genotype and environment, as evidenced by the fluctuations in concentration; for instance, genotypes like G1 and G6 show higher linalool accumulation during the November sowing periods, whereas petroselinic acid levels exhibit distinct patterns across the March sowings, particularly in G5. Ultimately, these figures demonstrate that the chemical output of the species is highly plastic, suggesting that selecting the appropriate genotype (such as G1 or G6) and optimizing the sowing date are essential strategies for maximizing the yield of specific bioactive compounds.

**Figure 3:**
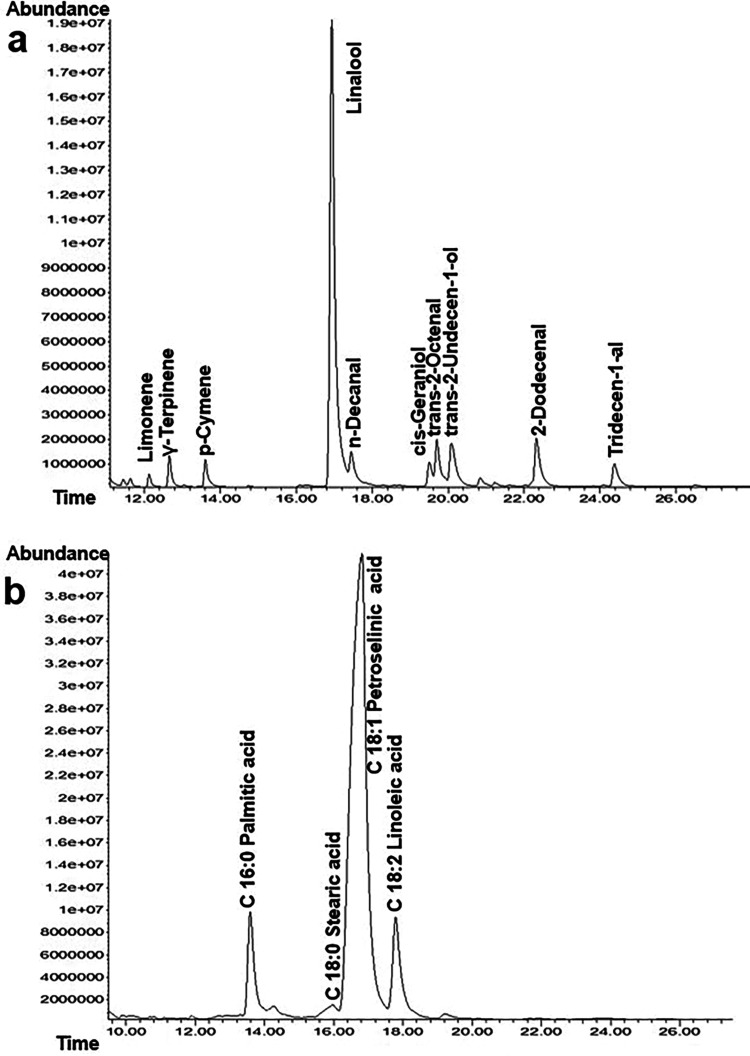
Representative GC-MS chromatograms illustrating the chemical profiles of (a) essential oil constituents and (b) fatty acid composition.

**Figure 4:**
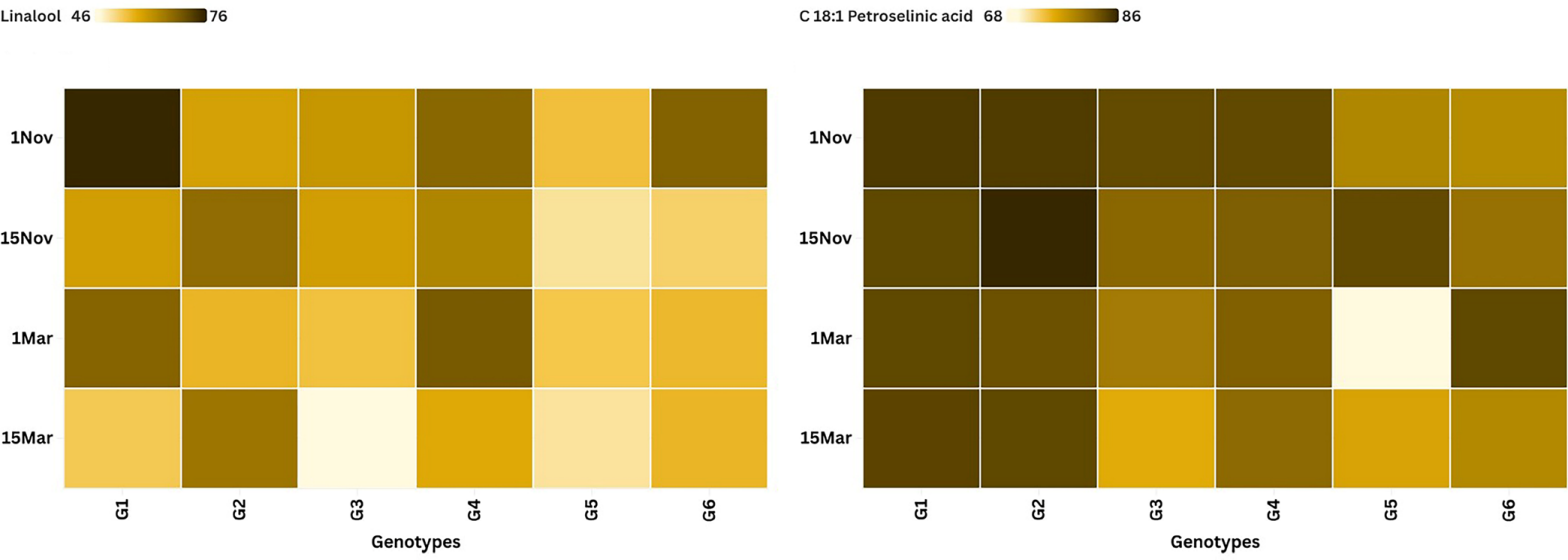
Heat map of essential oil and fixed oil components according to different sowing dates and genotypes (G1: Erba, G2: Gamze, G3: Irak, G4: Kudret, G5: Mardin, G6: Pelmus).

### 3.4 Multivariate Analysis (Correlation and PCA)

The Pearson correlation analysis is presented in Figure 5. Significant positive correlations were observed among the yield components. Specifically, seed yield (SY) exhibited a strong positive correlation with plant height (PH), number of branches (NB), number of umbels (NU), and seed weight per plant (SW). These findings suggest that genotypes with vigorous vegetative growth and a higher number of generative structures tend to produce higher seed yields. Regarding quality traits, distinct relationships were identified within the essential oil components. Linalool (Lin), the major component of coriander oil, showed a strong negative correlation with several aldehydes, including n-decanal, 2-dodecenal, and tridecen-1-al (as indicated by the negative coefficients in the matrix). This inverse relationship suggests a trade-off in the biosynthesis pathway where an increase in linalool content is associated with a decrease in these minor aldehyde components. A key biochemical trend identified in this study is the remarkable stability of the MUFA skeleton, primarily petroselinic acid, which remained the dominant component regardless of the dramatic shifts in sowing dates. However, we observed a highly significant inverse relationship between petroselinic (C18:1) and linoleic (C18:2) acids. This suggests a compensatory trade-off in the fatty acid desaturation pathway, where environmental timing influences the degree of unsaturation without compromising the core monounsaturated nature of the oil. These findings, supported by PCA, distinguish genotypes like G1 (Erba) as superior candidates for maintaining high-quality oil profiles under fluctuating Mediterranean conditions.

**Figure 5:**
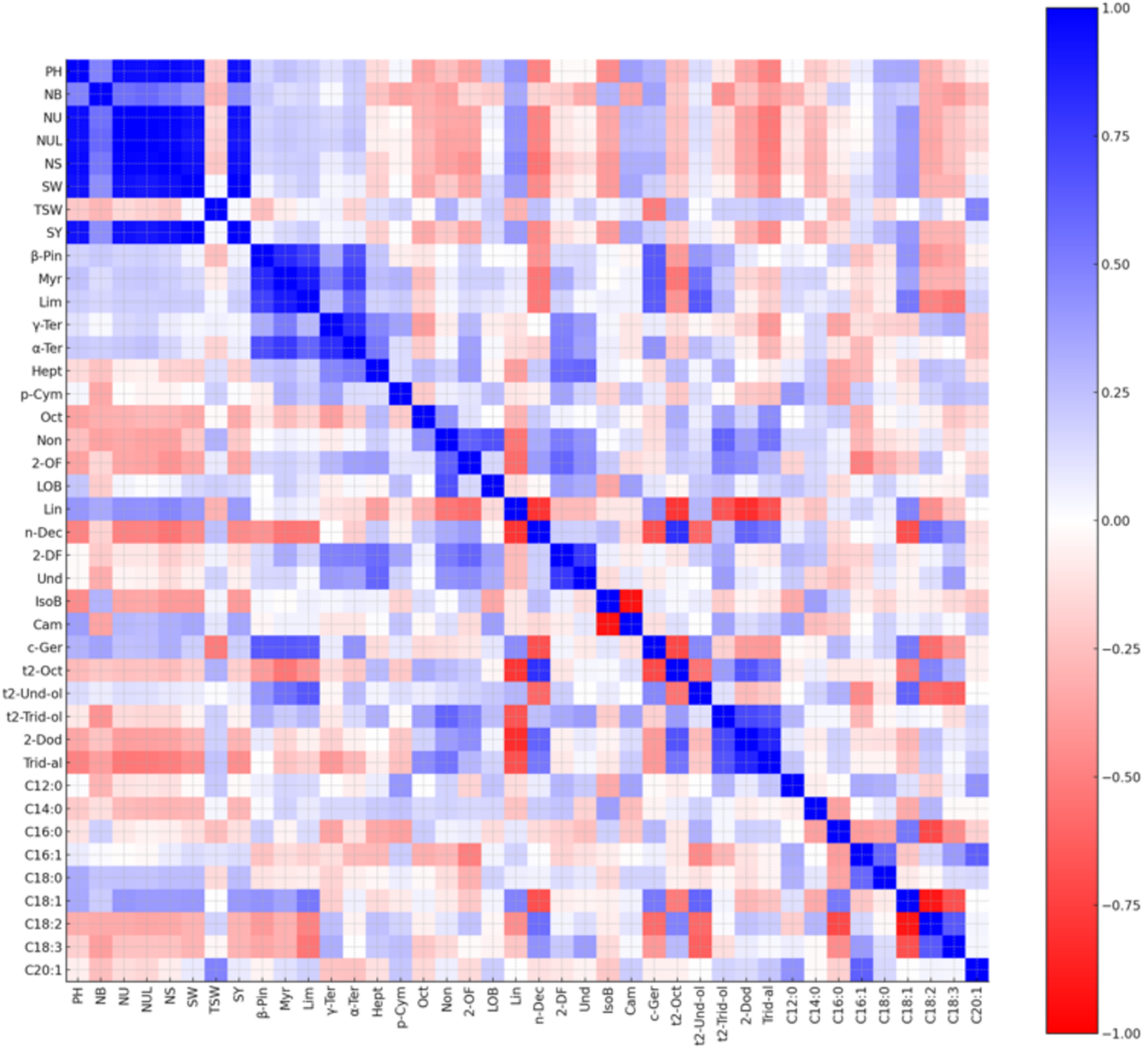
Heat map of the correlation matrix representing the relationships between agronomic parameters and quality traits: Plant height (PH), number of branches (NB), number of umbels (NU), number of umbellets (NUL), number of seeds (NS), seed weight per plant (SW), thousand seed weight (TSW), seed yield (SY), β-pinene (β-Pin), myrcene (Myr), limonene (Lim), γ-terpinene (γ-Ter), α-terpinene (α-Ter), heptanal (Hept), p-cymene (p-Cym), octanal (Oct), nonanal (Non), 2-octylfuran (2-OF), linalool oxide B (LOB), linalool (Lin), n-decanal (n-Dec), 2-decylfuran (2-DF), undecanal (Und), isoborneol (IsoB), camphor (Cam), cis-geraniol (c-Ger), trans-2-octenal (t2-Oct), trans-2-undecen-1-ol (t2-Und-ol), trans-2-tridecen-1-ol (t2-Trid-ol), 2-dodecenal (2-Dod), tridecen-1-al (Trid-al), C12:0 lauric acid (C12:0), C14:0 myristic acid (C14:0), C16:0 palmitic acid (C16:0), C16:1 palmitoleic acid (C16:1), C18:0 stearic acid (C18:0), C18:1 petroselinic acid (C18:1), C18:2 linoleic acid (C18:2), C18:3 linolenic acid (C18:3), and C20:1 gondoic acid (C20:1).

The PCA biplot, accounting for 39.6% of the total variance, reveals a clear distinction between sowing times along the first principal component (PC1) (Figure 6). November (autumn) sowings were strongly characterized by superior agronomic performance, evidenced by the tight clustering of yield-related traits (PH, NB, SW, and NS) and major monoterpenes (limonene and myrcene) in the positive PC1 region. Additionally, a strong negative correlation was observed between petroselinic acid (C18:1) and polyunsaturated fatty acids (C18:2 and C18:3), with the latter being more prominent in March (spring) sowings. Overall, genotypes G1 (Erba) and G2 (Gamze) planted in November displayed the most favorable profile, combining a high seed yield with elevated essential oil and monounsaturated fatty acid contents.

**Figure 6:**
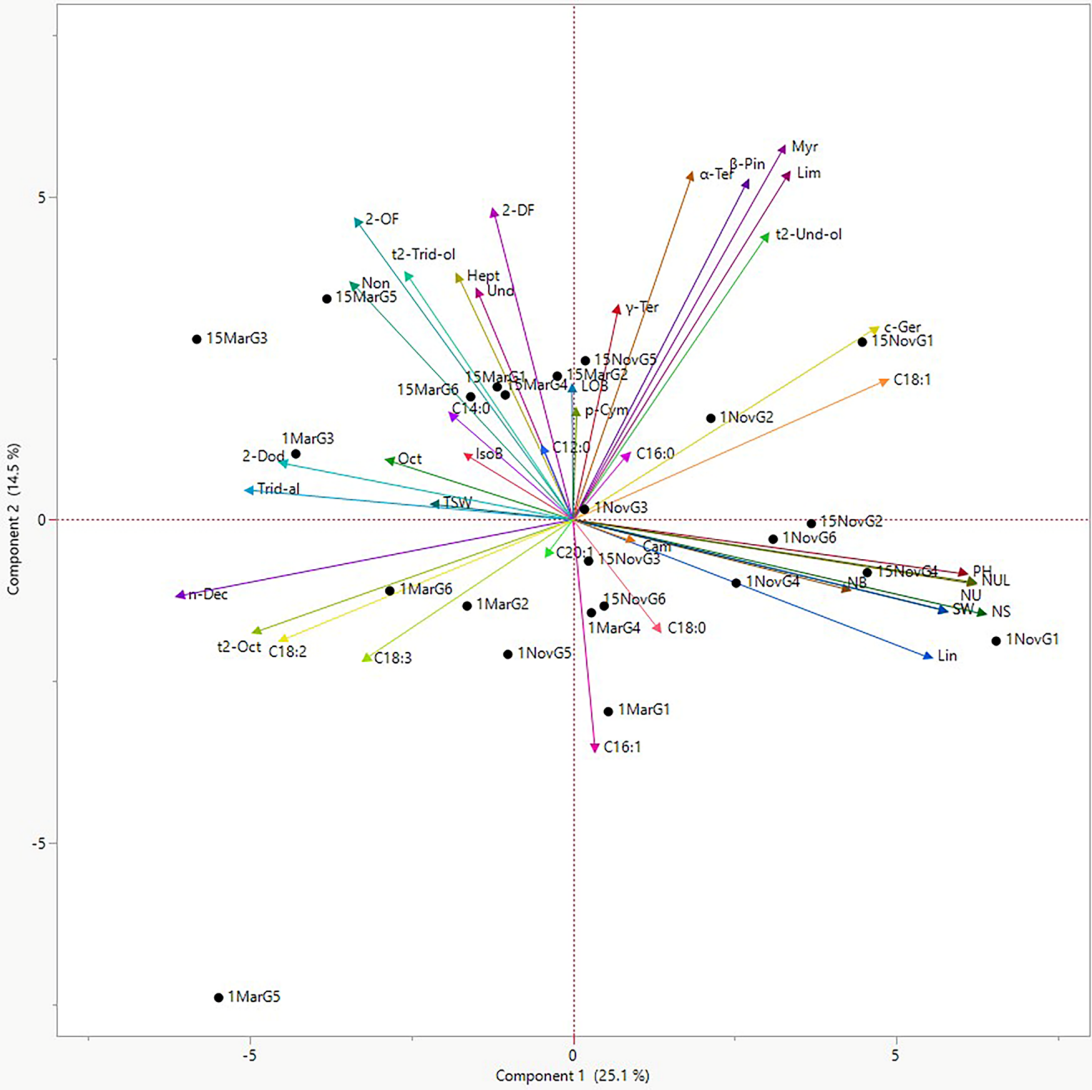
PCA on correlations of the agronomic traits, essential oil components, and fatty acids: G1 (Erba), G2 (Gamze), G3 (Irak), G4 (Kudret), G5 (Mardin), G6 (Pelmus), plant height (PH), number of branches (NB), number of umbels (NU), number of umbellets (NUL), number of seeds (NS), seed weight per plant (SW), thousand seed weight (TSW), seed yield (SY), β-pinene (β-Pin), myrcene (Myr), limonene (Lim), γ-terpinene (γ-Ter), α-terpinene (α-Ter), heptanal (Hept), p-cymene (p-Cym), octanal (Oct), nonanal (Non), 2-octylfuran (2-OF), linalool oxide B (LOB), linalool (Lin), n-decanal (n-Dec), 2-decylfuran (2-DF), undecanal (Und), isoborneol (IsoB), camphor (Cam), cis-geraniol (c-Ger), trans-2-octenal (t2-Oct), trans-2-undecen-1-ol (t2-Und-ol), trans-2-tridecen-1-ol (t2-Trid-ol), 2-dodecenal (2-Dod), tridecen-1-al (Trid-al), C12:0 lauric acid (C12:0), C14:0 myristic acid (C14:0), C16:0 palmitic acid (C16:0), C16:1 palmitoleic acid (C16:1), C18:0 stearic acid (C18:0), C18:1 petroselinic acid (C18:1), C18:2 linoleic acid (C18:2), C18:3 linolenic acid (C18:3), and C20:1 gondoic acid (C20:1).

## 4 Conclusion

This study elucidates that the agronomic and phytochemical architecture of coriander is not merely a product of its genetic makeup but is governed by a critical synchronization between sowing windows and thermal accumulation (GDD). Our findings move beyond the observation of annual yield fluctuations, revealing that the Genotype × Environment interaction can be strategically leveraged to produce divergent raw materials from the same genetic stock. The research identifies a fundamental metabolic reprogramming in coriander essential oils induced by environmental timing; while early autumn sowings (Nov 01) maximize biomass and linalool-rich oil yield, late spring sowings trigger a shift toward aliphatic aldehydes (n-decanal and 2-dodecenal). This shift suggests that temperature-driven stress and rapid heat unit accumulation during the reproductive phase—quantified through GDD and Heat Use Efficiency (HUE)—act as a biological “switch” to target different industrial sectors. Consequently, the traditional view of spring sowing as a mere “yield loss” is challenged; instead, it is presented as a specialized production model for the niche perfume and cosmetic industries requiring “green” olfactory notes. From a breeding and selection perspective, the “Erba” genotype (G1) demonstrated the highest phenotypic stability and yield potential, making it a superior candidate for large-scale linalool production in Mediterranean-type climates. Furthermore, the observed inverse correlation between petroselinic and linoleic acids in the fixed oil fraction confirms that environmental conditions significantly modulate the fatty acid desaturation pathway. Ultimately, these results establish a dual-purpose cultivation roadmap that allows producers to strategically align coriander production with specific industrial requirements. To achieve maximum seed yield and produce linalool-dominant essential oils, early autumn sowing with stable genotypes like “Erba” is recommended to ensure optimal vegetative development before the reproductive transition. Conversely, for the production of aldehyde-rich specialty oils, late spring sowing windows should be utilized. This approach requires accepting lower biomass yields in exchange for high-value chemical plasticity specifically tailored to niche cosmetic and perfumery markets. By implementing this strategic framework, environmental variability—traditionally viewed as a cultivation risk—is transformed into a productive tool for chemical diversification, effectively aligning crop outputs with diverse market demands.

## Supplementary Material



## Notes

Correlation graph between meteorological variables and agronomic traits (Figure S1); growing degree days (°C·day) and heat use efficiency (kg seed/ha/°C·day) for seed yield under different sowing dates across the 2020–2021 and 2021–2022 seasons (Table S1); and monthly averages of daily evapotranspiration (mm), relative humidity (%), wind speed (m/s), and soil temperature at 5 cm depth (°C) for both growing seasons and the long-term 1995–2022 climatic averages at the experimental site (Table S2) (PDF)
