# Supplementary material for: Yield Performance and Oil Chemotype Variation of Coriandrum sativum L. Genotypes under Different Sowing Dates in a Mediterranean Climate
Source: ACS Omega. 2026 Mar 17;11(12):19658–71. doi: 10.1021/acsomega.5c13325 (PMC13044647; doi:10.1021/acsomega.5c13325)
Supplement: Supplementary file 1 [file ao5c13325_si_001.pdf]

Yield Performance and Oil Chemotype Variation of *Coriandrum sativum* L. Genotypes under Different Sowing Dates in a Mediterranean Climate

[1]Dua Ahmet Ali Ali, [1]Muzaffer Barut, [2]Ozlem Toncer, [3]Sengul Karaman, [1]Leyla Sezen Tansi*

[1] Department of Field Crops, Faculty of Agriculture, Çukurova University, 01330 Adana, Turkey

[2] Department of Field Crops, Faculty of Agriculture, Dicle University, 21280, Diyarbakir, Turkey

[3] Department of Biology, Faculty of Science and Letter, Kahramanmaras Sutcu Imam University, 46100, Kahramanmaras, Turkey

[*]Corresponding author: Muzaffer Barut

Mail: mbarut@cu.edu.tr

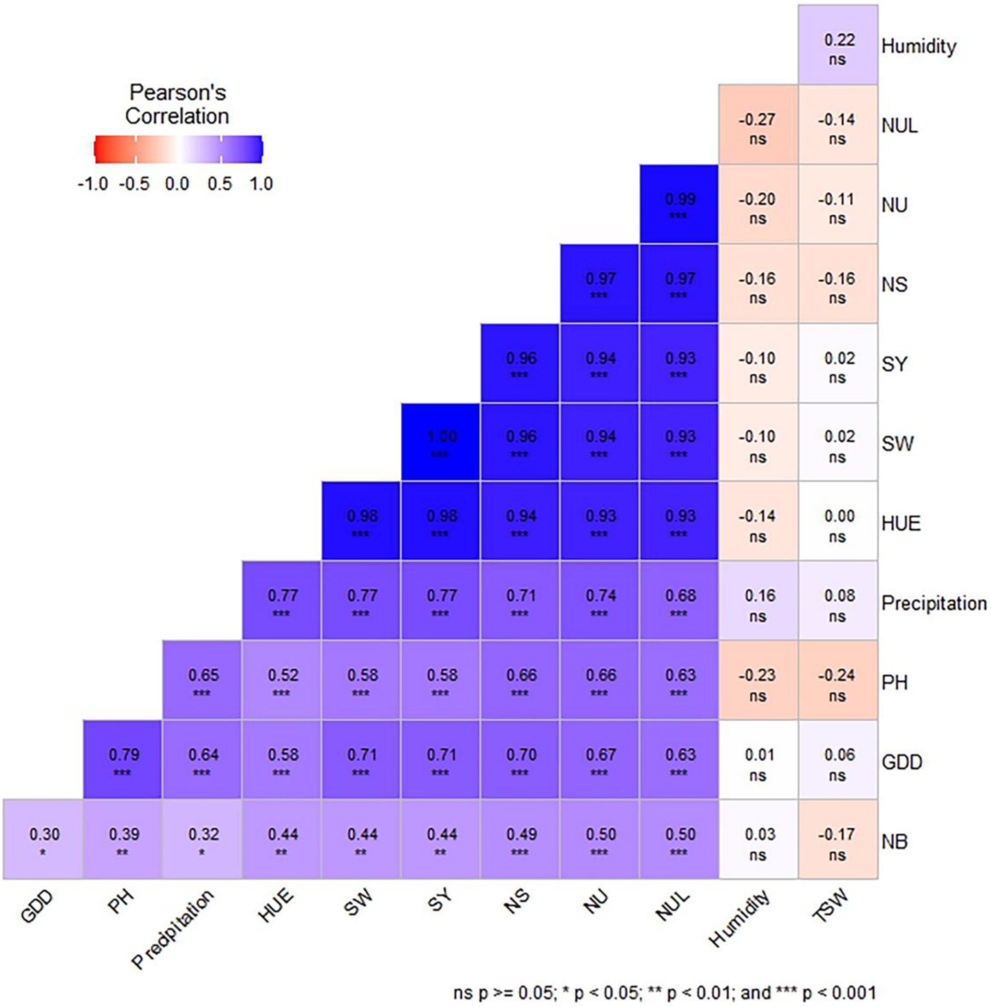

Figure S1. Correlation graph in terms of meteorological data and agronomic traits (GDD: Growing Degree Days; PH: Plant Height; HUE: Heat Use Efficiency; SW: Seed Weight per plant; SY: Seed Yield; NS: Number of Seeds; NU: Number of Umbels; NUL: Number of Umbellets; NB: Number of Branches; TSW: Thousand Seed Weight.)

Table S1. Growing Degree Days (GDD) and Heat Use Efficiency (HUE) for seed yield as influenced by different sowing dates during 2020–2021 and 2021–2022.

Factors	Growing Degree Days (GDD) (°C day)	Heat Use Efficiency (HUE) (kg seed/ha/deg. day)
**2020–2021**	1587.9	0.599
**01-Nov**	1861.4	0.723
**15-Nov**	1658.5	0.735
**01-Mar**	1379.6	0.524
**15-Mar**	1452.1	0.412
**2021–2022**	1674.0	1.808
**01-Nov**	1797.4	2.739
**15-Nov**	2158.1	2.233
**01-Mar**	1411.6	1.376
**15-Mar**	1328.9	0.883

Table S2. Monthly averages of daily evapotranspiration (ET_o), relative humidity, wind speed, and soil temperature during the 2020–2021 and 2021–2022 growing seasons compared to the long-term (1995–2022) climatic averages at the experimental site

**Years**	**Months**	**Monthly Average Daily Evapotranspiration (mm)**	**Monthly Average Relative Humidity (%)**	**Monthly Average Wind Speed (m/s)**	**Monthly Average Soil Temperature at 5 cm depth (°C)**
**2020-2021**	Nov.	4.18	59.50	1.28	17.23
	Dec.	3.40	63.90	1.36	12.54
	Jan.	2.74	63.65	1.31	11.06
	Feb.	3.25	61.43	1.30	12.58
	March	4.15	65.53	1.50	14.93
	Apr.	5.46	68.65	1.33	19.74
	May	8.20	64.87	1.43	25.87
	Jun.	10.87	67.25	1.77	31.43
	Jul.	12.72	67.99	1.77	35.78
**2021-2022**	Nov.	1.25	66.47	0.90	17.46
	Dec.	2.78	66.75	1.16	11.64
	Jan.	2.84	66.21	1.57	9.10
	Feb.	2.41	71.75	1.20	11.63
	March	0.10	58.55	1.36	12.29
	Apr.	6.17	57.15	1.41	21.21
	May	7.46	61.81	1.42	27.28
	Jun.	8.67	72.79	1.47	32.44
	Jul.	11.19	65.87	1.48	36.47
**Long Term (1995-2022)**	Nov.	3.19	64.14	1.05	16.36
	Dec.	2.23	69.44	1.23	11.06
	Jan.	2.31	68.71	1.34	9.33
	Feb.	2.69	67.68	1.35	11.19
	March	3.28	66.72	1.35	15.12
	Apr.	4.10	67.15	1.31	20.16
	May	5.30	66.51	1.25	25.94
	Jun.	6.46	68.70	1.34	31.70
	Jul.	6.81	71.62	1.38	35.71
